# Effect of the Interfiber Bonding on the Mechanical Behavior of Electrospun Fibrous Mats

**DOI:** 10.1038/s41598-020-64735-5

**Published:** 2020-05-07

**Authors:** Poorya Chavoshnejad, Mir Jalil Razavi

**Affiliations:** Department of Mechanical Engineering, State University of New York at Binghamton, Binghamton, New York 13902 USA

**Keywords:** Mechanical engineering, Mechanical properties, Biomedical engineering, Polymers

## Abstract

Electrospun fibrous mats, characterized by their large surface-to-volume ratios, have unique and beneficial properties for various applications. The micro or nanoscale architectures of these structures significantly affects the mechanical properties of the material. The lack of knowledge for predicting the mechanical behavior of electrospun fibrous mats may prevent applications utilizing the se mats from reaching their full potential. In this paper, we propose a new computational model to predict the mechanical behavior of an electrospun fibrous mat by considering its microstructure and the percentage of the cross-points that are bonded. The model of the electrospun mat with randomly distributed fibers is considered in uniaxial and biaxial tension. Three cases are studied: (1) no interaction in cross-points of intersecting fibers, (2) half of the cross-points are bonded, (3) all of the cross-points are bonded. The results show that along with the mechanical properties of individual fibers, the fusion bonding of fibers is a critical parameter for tuning the mechanical properties of the bulk material. In a predefined porosity, the interfiber fusion enhanced the stiffness of the mat by 60%, which is independent of the loading mode and the mechanical property of individual fibers. For all ranges of porosities, bonding increases the stiffness of the mat; however, the bonding is more effective at stiffening when the porosity of the mat is low.

## Introduction

Electrospinning is an advanced material processing method that uses high voltages to fabricate polymeric fibers with diameters ranging from 20 nm to 20 µm^1,2^. The electrospinning technique has four steps: (1) charging the Taylor cone with solution; (2) straight jet extrusion of the solution; (3) whipping the straight jet in the presence of an electric field that makes thinner jet flow; (4) solidification of the liquid jet on the target ground^2,3^. In this technique, polymer fibers are deposited into a grounded target using a high-voltage power supply. Prominent characteristics of the electrospinning mat (such as flexibility, simplicity, high surface area to volume ratio, controllable pore size and morphology, etc.) and also adjustable parameters that allow the creation of a mat with desired characteristics, broadens the applications of this technique. Flexible and porous electrospun mats with large surface-to-volume ratios are good candidates when permeability or absorptivity are needed. Electrospun fibrous mats have many applications such as filtration membranes, fiber reinforcements, catalytic supports, energy harvesting, photonic and electronic devices, drug delivery, and tissue scaffolding^2,4,5^. Using additives, such as nanoparticles, protein, and pigments, with the solution from electrospinning can provide specific characteristics for special applications^6–10^. To name a few, antibacterial nanoparticles, such as silver nanoparticles, can significantly enhance the inhibition of the mat against the growth of some bacteria^11^, pigment additives are used to fabricate colored mats^12–14^, and the photostability of the colored mat is improved by embedding the fibers in PDMS film^15^. Currently, much experimental data exists regarding the material characterization of fibers to control and tune the mechanical behavior of electrospun fibrous mats. However, it is a significant challenge to experimentally connect the behavior of bulk material to its non-trivial microstructure and the characteristics of constituent fibers^1^. It has been shown that the mechanical properties of an electrospun mat have a direct dependency on its constituent fibers’ diameter^16–19^, alignment^20^, porosity^21–23^, and bonding^24–26^. In contrast to continuum materials, the mechanical characterization of fibrous mats is not straightforward because of their complexity in porosity, microstructure, and the properties of their constituent fibers^24^. Despite research in the field, the characterization of the mechanical behavior of electrospun fibrous mats according to their microarchitecture remains a stimulating topic. Since the material properties of electrospinning mats are not relatively applicable, several techniques such as hot pressing^27^, cross linking^28,29^, and solvent vapor welding^30^ are provided to improve their mechanical features. The advantage of the solvent vapor technique is that only the cross-points of the fibers are welded together. Thus, the morphology and the porosity of the mat are maintained after the treatment^30,31^. Furthermore, unlike the cross-linking treatment, the solvent vapor technique is not detrimental to the environment^31,32^.

To overcome the difficulties associated with the mechanical characterization of electrospun fibrous mats, various multiscale modeling techniques link the behavior of the bulk material to the microstructure architecture^1^. Stylianopoulos *et al*.^33^ used a computational model to investigate the effect of fiber diameter and fiber orientation on the tensile properties of electrospun fiber meshes. They developed a multiscale modeling strategy and found that the microstructural architecture has a considerable effect on the tensile properties of the mat. Pai *et al*.^34^ proposed two quantitative microstructure-based (straight fibers and curved fibers) models to study the effect of fiber curvature on the elastic modulus of a nonwoven mesh. That study showed that along with porosity, the elastic modulus of fibers, the average fiber diameter, and the curvature of fibers are important factors that affect the mechanical properties of nonwoven mats. Rizvi *et al*.^35,36^ developed mathematical and statistical models to study the influence of microstructure on the mechanical behavior of fibrous materials. By using a probability density function, they showed that an increase in the flexibility of the fibrous material makes the material stronger. The statistical model revealed that the induced tension in the fibers on the matrix deformation is heterogeneous, and, therefore, the mechanical stimuli could be different at any location in the fibrous scaffold. Vatankhah *et al*.^37^ developed a neural network model to study the effect of composition, fiber diameter, and fiber orientation on the elastic modulus of the PCL/gelatin scaffold. They concluded that polymeric composition is the most important parameter affecting the elastic modulus of the scaffold. Depending on the ambient or physiological conditions, the diameter or orientation of fibers could be ranked at the second level.

**Figure 1:**
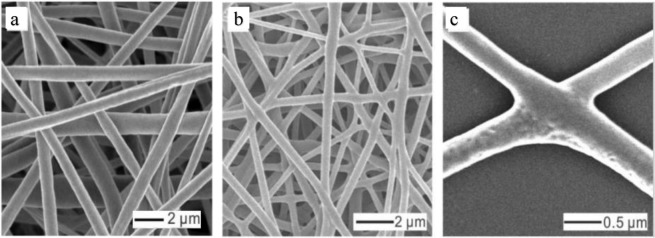
SEM images of the unbonded and bonded nanofiber mat. (**a**) image of the polycaprolactone (PCL) unbonded nanofibers; (**b**) image of the PCL bonded nanofibers; (**c**) cross-point of two intersecting fibers in a bonded PCL mat. The figure has been used from ref. ^30^. with permission.

Recently, among different computational methods, the finite element method (FEM) has been successfully used to understand the effect of the mechanical behavior of single fibers on the mechanical performance of fibrous mats^20,24,33,38–42^. Driscoll *et al*.^43^ developed a finite element model to determine the effect of fiber aspect ratio and orientation on the shear mechanics of oriented electrospun nanofibrous scaffolds. Results showed that the response of the scaffold in shear is affected by the fiber orientation and sample aspect ratio. A more uniform distribution of shear strain was observed in samples with a lower aspect ratio. Stylianopoulos *et al*.^20^ developed a finite element model to predict the mechanical behavior of electrospun cellulose acetate fiber meshes with random orientations. Their model estimated that changes in the fiber alignment might result in a 3.6 or 8.5-fold increase in the elastic modulus for moderately aligned and highly aligned meshes, respectively. They concluded that polymer concentration, fiber alignment, and solvent are the most important parameters affecting the mechanical and fluid transport properties of electrospun meshes. The deformation and damage behavior of a nonwoven mat made by thermal bonding of polypropylene fibers was analyzed by a finite element simulation^42^. It was found that the failure of the nonwoven mat results from the localization of damage initiation and propagation. Recently, Yin *et al*.^39,40^, by an analytical and finite element model, established the tensile constitutive relationship between fibers and mat. Their finite element model was developed for uniaxial and biaxial loading regimes and verified by biaxial tension experiments on the silk fibroin/polycaprolactone nanofibrous mats. The results of the studies confirmed that the constitutive relation and finite element model are able to satisfactorily explain the elastic-plastic tensile mechanical behaviors of the polymer. However, in most of the past studies, bonding (fusion) among intersecting fibers has not been included in the models. Fusion or welding at cross-points among fibers can be induced by thermal treatment^44–47^, solvent (or vapor) exposure^30,48^, and covalent cross-linking^28,49^. The fusion of intersecting fibers is an effective way to enhance the mechanical and electrical properties of a mat^2^. Li *et al*.^30^ by an experimental study showed that bonding among fibers remarkably increases the elastic modulus of a PCL nanofibrous mat. Figure 1 shows a PCL nanofiber mat before and after being exposed to vapor for inducing interfiber bonding.

Wei *et al*.^24^ developed a molecular dynamics (MD) model to predict the deformation of an electrospun nanofiber mat by considering the fusion among fibers and the van der Waals interaction. The results of this study showed that the interfiber fusion has a significant effect on the tensile strength of the mat. A higher number of fusion points increases the strength of the mat, and over-fusion may reduce the fracture energy. Therefore, ignoring the effect of fiber-fiber interaction on the mechanical properties of fibrous mats is not reasonable^24,42^. Accordingly, the absence of a computational framework for fused (welded) electrospun fibers limits their applications to less than their full potential regardless of their advantages.

In this study, we propose a new computational model to predict the mechanical behavior of a fibrous mat by taking the characteristics of randomly distributed constituent fibers and their interfiber bonding into account. This is a new approach that considers the bonding among intersecting fibers in a multiscale model and not just on a nanoscale^24^. The developed parametric model allows for the controlling of the diameter and material property of individual fibers as well as the percentage of bonding among intersecting fibers. The model is simulated and analyzed for both uniaxial and biaxial tension modes.

## Results and Discussions

To build the mat in a square cell, the fibers are laid down randomly in amounts needed to control porosity, and are crossed with each other at different angles. Each junction is called a cross-point, and the stiffness of the mat varies with the proportion of cross-points that are bonded, or fused together. We examined three levels of fiber bonding. In 0% bonding (unbonded), none of the fibers were welded together and the fibers could slide over each other at the cross-points. If half of the cross-points are fused, the mat is 50% bonded, and a mat where every cross-point is fused is termed 100% bonded. We examined each of these three levels with the mat under uniaxial or biaxial tension (see Methods section for the details of the diameter and elastic modulus of fibers, porosity and implemented boundary conditions in the models).

**Figure 2:**
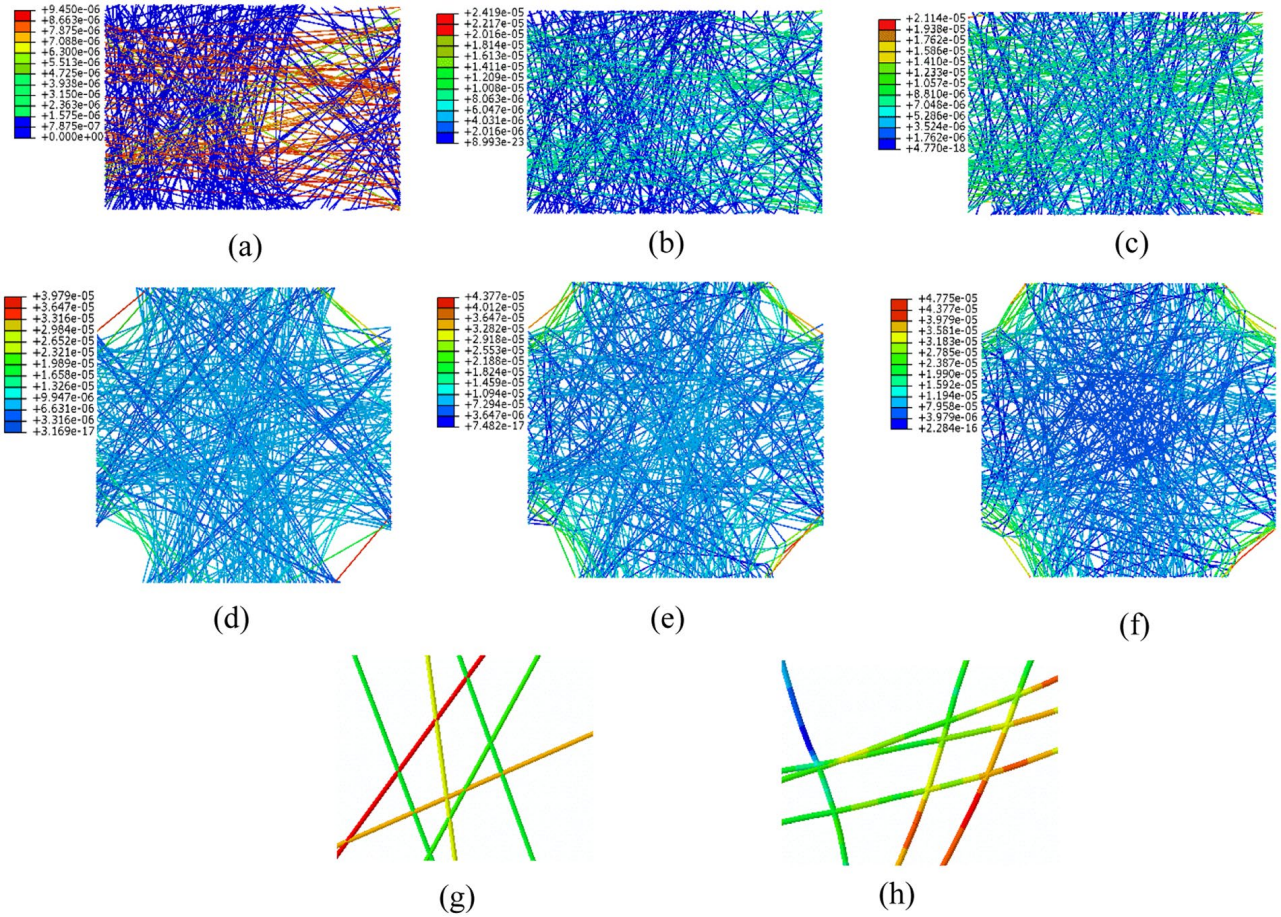
Von Mises stress (N/µm^2^) distribution in a 45% stretched fibrous mat under uniaxial and biaxial tension. (**a**) uniaxial tension with 0% interfiber bonding; (**b**) uniaxial tension with 50% interfiber bonding; (**c**) uniaxial tension with 100% interfiber bonding among intersecting fibers; (**d**) biaxial tension with 0% interfiber bonding; (**e**) biaxial tension with 50% interfiber bonding; and (**f**) biaxial tension with100% bonding among intersecting fibers. Blue to red shows higher stresses. (**g**) a zoomed-in image for stress distribution in 0% bonding fibers; (**h**) a zoomed-in image for stress distribution in 100% bonded fibers. The porosity is 25% in all models.

Figure 2 shows the stress distribution of the mat for all three cases under the 45% uniaxial and biaxial tensile strains. The pattern of random distribution of fibers in all of the models is the same, and only the applied strain mode and the percentage of bonding are different. In case (a) in Fig. 2, all cross-points between fibers are free to move without interaction. It can be seen that fibers that reach both the left and right sides of the mat carry the load, and the other fibers do not contribute. Thus, the induced stress in most of the fibers is zero. Moreover, the displacement of fibers that do not reach the right edge is zero. In case (b), 50% of fibers’ cross-points are bonded, and the rest have no interaction. In this case, the bonded cross-points are randomly selected from all available intersections. In comparison with case (a), more fibers carry the load because even if a fiber does not reach the right edge, it will stretch through its connections to other fibers. Consequently, as more fibers are load-bearing, case (b) requires more force to be stretched. In case (c), all cross-points are bonded. In this case, all fibers carry the load, which makes the case (c) the stiffest among all cases under uniaxial tension. However, fibers that reach from the left to right edges experience more stress than fibers that do not span the mat’s width.

Figure 2(d–f) show the stress distribution in a mat under 45% biaxial tension. In case (d), there is no interaction between fibers. In contrast to case (a), all fibers are load-bearing even without interfiber bonding, because the mat in biaxial tension is stretched in two directions. Figure 2(e,f) show the stress distribution in cases with 50% and 100% bonding in the cross-points, respectively. In case (f), the mat reaches higher stresses in comparison with cases (d) and (e), because there are more interactions among fibers. Figure 2(g,h) show zoomed-in images of the stress distribution in a few intersecting fibers for 0% and 100% bonding cases, respectively. The induced stress in two cases shows the effect of bonding in the cross-points of the fibers. In the unbonded case stress remains the same before and after the cross-point, while in the bonded case stress varies before and after cross-point. Figure 2 also shows that the mat under biaxial tension reaches higher stresses in all cases in comparison with the mat under uniaxial tension.

In each of the following cases, results were obtained to reduce the randomness effect by running five random models for each case. The reported results are the average of the outputs from the five models with different fiber distributions.

### Fibrous mat under uniaxial tension

**Figure 3:**
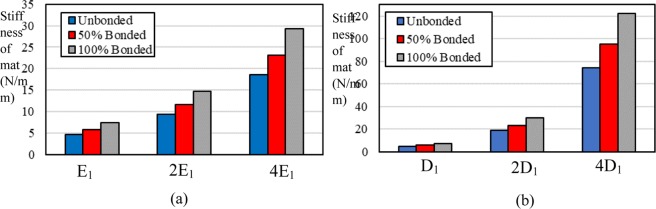
(**a**) Effect of the elastic modulus of the single fiber on the stiffness of the mat in uniaxial tension. E_1_ is equal to 23.40 MPa. (**b**) effect of the diameter of the single fiber on the stiffness of the mat in uniaxial loading. D_1_ is equal to 0.7 µm.

Figure 3(a) shows how the stiffness of a mat varies with increases in the number of bonded fibers in the three levels of elastic moduli for the fibers. The stiffness is defined as the slope of the force-displacement curve for the stretched mat. The elastic modulus E_1_ and Diameter of the Fiber D_1_ in Fig. 3 are equal to 23.40 MPa and 0.70 µm, respectively. Results show that the stiffness of the mat generally has a linear dependency on the elastic modulus of the fibers for all unbonded cases, and both of the bonded cases as well. The stiffness of the mat for the unbonded case is equal to 4.68, 9.29, and 18.57 N/mm, when the elastic modulus of the fibers is set to 23.40, 46.80, and 93.60 MPa, respectively. Applying 50% bonding increases stiffness to 5.78, 11.57, 23.15 N/mm. An increase in bonding percentage leads to a stiffer mat, and the 100% bonded case has the most stiffness. The results interestingly show that the stiffness ratio between mats with the same bonding percentage and different elastic modulus of the fibers is almost independent of the elastic modulus of the fibers. As an example, the stiffness ratio between mats with 0% and 100% bonding is 0.638, 0.633, and 0.634 when the elastic modulus of the fibers is set to 23.40, 46.80, and 93.60 MPa, respectively.

Figure 3(b) shows the effect of the fiber diameters on the stiffness of the mat under uniaxial tension for different bonding percentages. The increase in the diameter of the fibers profoundly enhances the stiffness of the mat in a nonlinear manner. As an example, for a case with 100% bonding, the stiffness increases 16.6 times when the diameter quadruples. This result is expected because the induced force in a stretched single fiber is proportional to the square of its diameter. Similar to the variation in the elastic modulus of the fibers, the stiffness ratio between unbonded, 50% bonding, and 100% bonding remains roughly the same for all of the fiber diameters. For instance, the equivalent stiffness ratio between cases with 0% and 100% bonding and diameter of 0.70 µm and 2.80 µm is equal to 0.638 and 0.610, respectively.

**Figure 4:**
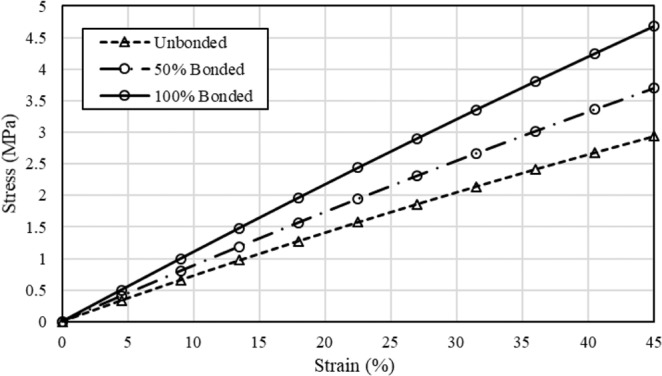
Effect of the bonding between intersecting fibers on the mechanical behavior of the mat under uniaxial tension.

Figure 4 shows the stress-strain curve of the mat for the unbonded, 50% bonded, and 100% bonded cases. As the percentage of bonding increases, the induced stress in the mat increases as well. We consider the elastic modulus of the mat as the slope of the stress-strain curve in both simulation and experiment from ref. ^30^. Simulation results show that the bonding (welding in the experiment) increases the elastic modulus of the mat close to 1.57-fold, which is in the same order with the experimental results showing a 1.96-fold increase^30^. We speculate that the difference between simulation and experimental results is attributed to the variation of the elastic modulus of a single fiber during the vapor treatment. This variation has not been included in FE models, because it has not been quantified in these experiments^30^. Fig. 2(e,f) from ref. ^30^, clearly shows that the treatment changes the material property of the fibers and most likely causes them to increase in stiffness. Another possible reason for the varying results in the simulation and experiment is the different porosities used in each. The porosity in the simulations is 25%, while there is no information regarding the porosity in the experiment^30^. Later, we will show that the effectiveness of bonding is dependent on the porosity of the mat. Our results also are in good agreement with the result of another experimental study^44^, which used thermal treatment to improve the biomechanical properties of an electrospun PCL scaffold. The extracted result from the stress-strain curve in the elastic region from ref. ^44^. shows that the welding between fibers increases the elastic modulus of the mat close to 1.75-fold. To obtain more accurate results, the porosity and the material behavior of the fibers are needed.

### Fibrous mat under biaxial tension

**Figure 5:**
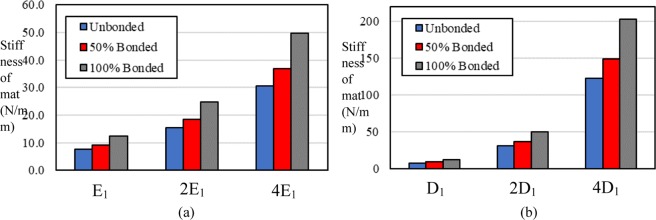
(**a**) Effect of the elastic modulus of the single fiber on the equivalent stiffness of the mat under biaxial tension and with different bonding percentage. E_1_ is equal to 23.40 MPa. (**b**) effect of the diameter of the single fiber on the equivalent stiffness of the mat under biaxial tension for different bonding percentages. D_1_ is equal to 0.70 µm.

Figure 5(a) shows the effect of the elastic modulus of the fibers on the stiffness of the mat under biaxial tension for different bonding percentages. The increase in the elastic modulus of the fibers increases the stiffness of the mat. The elastic modulus E_1_ and Diameter of the Fiber D_1_ in Fig. 3 are equal to 23.40 MPa and 0.70 µm, respectively. Similar to the uniaxial tension, the stiffness has a linear relation with the elastic modulus of the fibers. By doubling the elastic modulus of the fibers from 23.40 MPa to 46.80 MPa, the stiffness of mat increases from 12.44 to 24.9 N/mm for the 100% bonded case. As expected, similar to the uniaxial tension, the bonded mat has the highest stiffness among all cases. While the difference between stiffnesses of unbonded, 50% bonded, and 100% bonded cases increases as the elastic modulus of the fibers increases, the stiffness ratio of each of the two cases remains roughly the same. For instance, the stiffness ratio for unbonded and 100% bonded cases with the elastic modulus of single fiber as 23.40 MPa and 93.60 MPa is equal to 0.617 and 0.616, respectively.

Figure 5(b) shows the effect of the fiber diameter on the stiffness of the mat under biaxial tension for different bonding percentages. By comparing Fig. 5(a,b), it is clear that the diameter of the fibers has a more significant effect on the stiffness of mat than the elastic modulus. In contrast with the elastic modulus, the relation between the diameter of the fibers and the stiffness of the mat is nonlinear. Similar to the previous results, while the diameter of the fibers increases, the stiffness ratio between unbonded, 50% bonded, and 100% bonded cases remains the same. For instance, the stiffness ratio between unbonded and 100% bonded cases for models with diameters of 0.70 and 2.80 µm is equal to 0.618 and 0.605, respectively.

**Figure 6:**
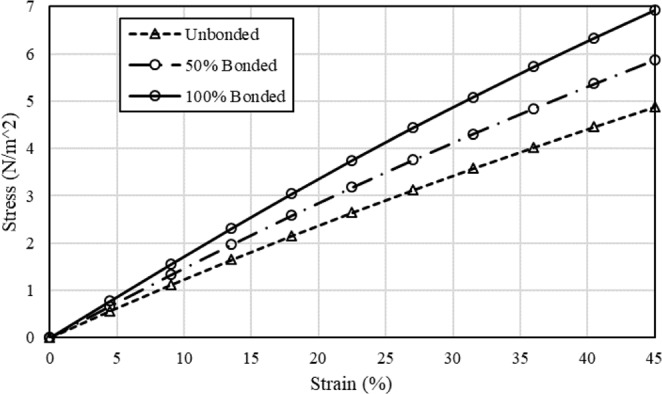
Effect of the bonding between intersecting fibers on the mechanical behavior of the mat under biaxial tension.

Figure 6 shows the dependence of the elastic modulus of the mat to the percentage of the bonding in cross-points. Similar to the uniaxial case, bonding of the intersecting fibers increases the elastic modulus of the mat. A comparison between Figs. 4 and 6 shows that for the same strain, higher stress is induced in the mat under biaxial tension than under uniaxial tension.

**Figure 7:**
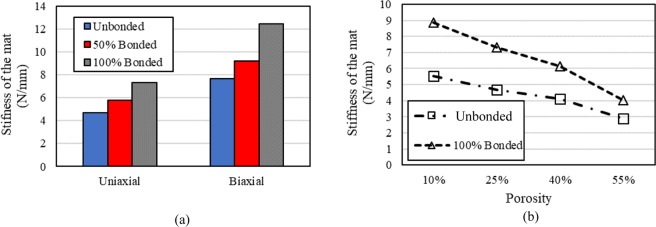
(**a**) Effect of the bonding on the stiffness of mat under uniaxial and biaxial tension in 25% porosity; (**b**) effect of the bonding on the stiffness of mat for different porosities in uniaxial tension.

Figure 7(a) shows the stiffness of the mat under uniaxial and biaxial tension for unbonded, 50% bonded, and 100% bonded cases. In all cases, the stiffness under biaxial tension is significantly higher than under uniaxial tension. The reason is that in uniaxial tension, the fibers that have been projected between left and right boundaries mostly carry the load. The rest of the fibers in the unbonded condition are stationary, and fibers in 50% bonding and 100% bonding act as the connective fibers and increase stiffness. Conversely in biaxial tension, fibers are stretched in two perpendicular directions and they all carry the load. Therefore, the effect of the bonding is more profound in biaxial tension, and the mat shows more resistance against displacement. It is interesting that in all cases and the two tension modes, the stiffness ratio between 100% bonded and unbonded cases is close to 1.6. Therefore, the interfiber bonding enhances the stiffness of the mat by 60%, which is not dependent on the tension modes, and the diameter, or elastic modulus of the fibers. Figure 7(a) indicates that increasing bonding percentage from 50% to 100% in both uniaxial and biaxial loading conditions considerably increases the stiffness of the mat. This result is in contradiction with the results of ref. ^24^, which indicates that both full and half fusions exhibit a slight difference in elastic modulus and tensile strength. The mentioned study has not discussed the reason for this peculiar observation.

Figure 7(b) shows the effect of bonding on the stiffness of the mat for different porosities in uniaxial tension. The porosity is the ratio of porous area to the total area of the mat. For all ranges of the porosities, bonding increases the stiffness of the mat. However, the ratio of stiffness for 100% and 0% bonding increases as the porosity decreases. In other words, the interfiber bonding is more effective when the porosity of the mat is low. The stiffness ratio for the cases with 100% and 0% bonding in 10%, 25%, 40%, and 55% porosity is 1.59, 1.56, 1.48, and 1.39, respectively. Another observation is that the difference between the stiffness ratios decreases as the porosity decreases. Therefore, the difference between the stiffness ratio for the cases with 55% and 40% porosity is more significant than the case with 25% and 10% porosity. These results show that the porosity, diameter and elastic modulus of the fibers along with the bonding percentage can tune the mechanical properties of the fibrous mats for different applications. In this model, we only considered randomly distributed fibers and did not assess the effect of fiber orientation. Nevertheless, as discussed in the introduction, fiber alignment is another critical parameter in tuning the mechanical properties of a fibrous mat^20^.

## Conclusion

The effect of the bonding among intersecting fibers on the stiffness of the mat has been rarely discussed in literature. Therefore, in this paper, we studied the effect of the interfiber bonding on the mechanical behavior of a fibrous mat. Two-dimensional nonlinear finite element models were created by randomly distributing the amount of fibers needed to achieve the requested porosity. We analyzed three cases with different percentages of bonding under uniaxial and biaxial tension. The results of the study show that the increase in the elastic modulus of the single fiber linearly enhances the stiffness of the mat. In contrast, the relation between the diameter of the single fiber and the stiffness of the mat is nonlinear and parabolic. Therefore, the diameter of the fibers is the dominant factor for controlling the stiffness of the mat. Results interestingly show that bonding in all cross-points of the intersecting fibers increases the stiffness of the mat 1.56 times, independently from the material properties and loading conditions. The developed models can predict with high accuracy the mechanical behavior of a fibrous mat according to the percentage of interfiber bonding, or by an inverse method, it is possible to predict the elastic modulus of an individual fiber according to the mechanical behavior of the mat. The latter, from the experimental view, is a challenging task in nanoscale. Therefore, computational models with high accuracy could be a prominent alternative to the expensive and time-consuming experiments for characterizing the mechanical properties of an individual fiber. Similar to other computational studies, there are some limitations in this study and thus future studies should continue to refine relationships investigated in the present study. An elastic behavior was used for the fibers, as only we considered the elastic region of the experiment. In higher strains, the fibers typically show an elastic-plastic behavior. Therefore, to find the ultimate strength of the mat, an elastic-plastic behavior should be used for the fibers. The friction force among contacting fibers also has not been considered in the models. In reality, there is slippage between the unbonded contacting fibers. Also, it should be mentioned that the fibers are considered to be straight lines without curvature and all of the parameters, such as the fiber diameter, bonding in cross-points, and the mechanical properties of the fibers, are assumed to be ideal. Thus, some parameters that affect the mechanical properties of the mat, such as thickening (beads), fiber agglomeration, ribbons and round fibers, are neglected. Studying the bonding effect with the curved fibers could be an interest for future studies.

## Materials and Methods

### Finite element model

**Figure 8:**
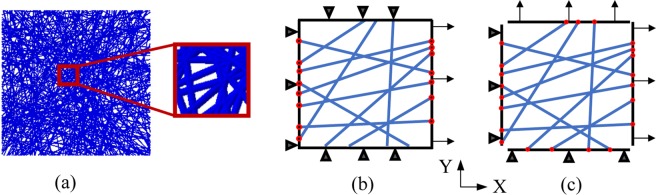
(**a**) Developed finite element model to mimic the small piece of a fibrous mat, (**b**) schematic of the boundary conditions for uniaxial tension, (**c**) schematic of boundary conditions for biaxial tension.

To study the effect of the interfiber bonding and microarchitecture characteristics on the mechanical behavior of a mat, a 2D non-linear finite element (FE) model was developed according to the experimental study of Li *et al*.^30^. The size of the square model was set as $100\times 100\,{\mu m}$. Since the porosity of the mat has not been mentioned in ref. ^30^, we arbitrarily assumed the porosity of the mat to be 25% in the FE model. The porosity is defined as the ratio of porous area to the total area of the mat. However, to suppress the porosity effect, we changed the elastic modulus of the fibers the amount necessary to produce a similar stress-strain curve of the experiment. Fibers are generated with random positions and orientations in such a way that the total surface area occupied by fibers reached 75% of the mat’s area^39,50^. Thus, the porosity of the mat is 25%. Fibers are modeled with beam element (B21), which is a common element used for fiber modeling^40,51^, in Abaqus commercial software. The diameter of the fibers is set to $700{nm}$ which is the average diameter of the fibers used in the experiment^30^. An auxiliary code was developed to control the bonding percentage between the intersecting fibers. Bonding means that at the fused (welded) cross-points, there is no relative motion between intersecting fibers. Considering the percentage of bonding in the cross-points, three cases were considered: (1) 0% bonding (unbonded), (2) 50% bonding, and (3) 100% bonding among intersecting fibers. In the first case, all the cross-points of the fibers are free to move independently. In the second case, half of the cross-points of the fibers in the mat are bonded together and the other half are free. To do so, all cross points are identified first, and then 50% of them are randomly selected by a script to add the tie constrain in the selected cross points. The other half of the cross points are left free without any interactions. In the last case, all the cross-points of intersecting fibers are bonded together in the mat. Figure 8(a) shows a finite element model that was created with random positions and orientations of the fibers. Figure 8(b,c) illustrate the boundary conditions of the uniaxial and biaxial tension in the model, respectively. For the uniaxial tension, the model is fixed from the left edge through the “X” axis and stretched from the right edge. A symmetric boundary condition is applied on both the top and bottom edges. For the biaxial tension, fibers are coupled together at all edges. The model is fixed in the “X” direction from the left edge and fixed in the “Y” direction from the bottom edge. The same displacement is applied for both right and top edges.

**Figure 9:**
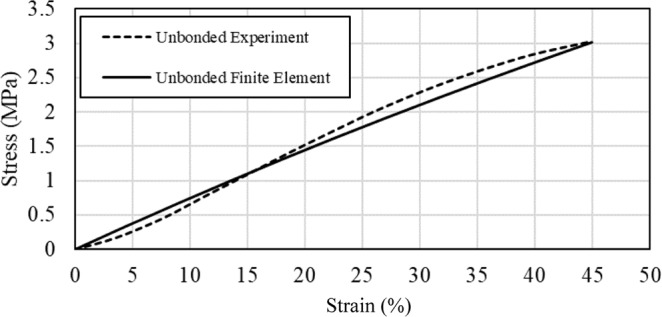
Experimental data is used to find the elastic modulus of a single fiber. The elastic modulus of a single fiber is varied as far as the stress-strain curve of the mat in FE model to be similar to the experiment. Experimental data is from ref. ^30^.

The material of fibers is assumed to be isotropic and elastic. Figure 9 shows how experimental data for unbonded fibers from^30^ was used to find the elastic modulus of an individual fiber. Ten random models were created and for each case, the elastic modulus of the fibers was found according to the same criterion that the produced stress-strain curve by the model. This was done to ensure the mat would be similar to the elastic region of the experimental curve. The elastic modulus of an individual fiber is the average of the obtained elastic moduli of ten cases. By this method, the elastic modulus of an individual fiber was found to be $23.40{\rm{MPa}}$. This value for the elastic modulus of the fibers was used as the base elastic modulus in all models of this study. The Poisson’s ratio was assigned as $0.5$ to mimic the incompressibility of the fiber’s material.

## Data Availability

Data produced and analyzed during this study are available from the corresponding author on request.
